# Obesity-Related Hypertension in Children

**DOI:** 10.3389/fped.2017.00197

**Published:** 2017-09-25

**Authors:** Tammy M. Brady

**Affiliations:** ^1^Division of Pediatric Nephrology, Johns Hopkins University School of Medicine, Baltimore, MD, United States

**Keywords:** pediatrics, blood pressure, cardiovascular disease, adiposity, adipocyte, adipose tissue dysfunction

## Abstract

Obesity and hypertension have both been on the rise in children. Each is associated with increased cardiovascular disease risk and both track into adulthood, increasing the prevalence of heart disease and related morbidity and mortality. All children should be screened for hypertension, but children with comorbid obesity may not only particularly benefit from the screening but may also prove the most challenging to screen. Increased arm circumference and conical arm shape are particularly problematic when attempting to obtain an accurate blood pressure (BP) measurement. This review focuses on the unique aspects of hypertension evaluation and management in the child with comorbid obesity. Specific traditional and non-traditional risk factors that may contribute to elevated BP in children with obesity are highlighted. Current proposed pathophysiologic mechanisms by which obesity may contribute to elevated BP and hypertension is reviewed, with focus on the role of the sympathetic nervous system and the renin–angiotensin–aldosterone system. This review also presents a targeted treatment approach to children with obesity-related hypertension, providing evidence for the recommended therapeutic lifestyle change that should form the basis of any antihypertensive treatment plan in this population of at-risk children. Advantages of specific pharmacologic agents in the treatment of obesity-related hypertension are also reviewed.

## Introduction

Since 1980, the prevalence of obesity among children and adolescents has almost tripled. Current estimates suggest that approximately 17% of children 2–19 years of age are obese. This percentage equates to 12.7 million children in the United States (1). These data are more striking when you include those who are overweight: 30%, or 25 million, US children are overweight/obese.

While these statistics are remarkable in their own right, the implications are particularly concerning. Children with obesity are at a significantly increased risk for cardiovascular disease (CVD): they have higher systolic and diastolic blood pressure (BP) and greater evidence of dyslipidemia and insulin resistance (2). In fact, 70% of obese children have at least one CVD risk factor, and 39% have two or more (3). These CVD risk factors, along with obesity, are not only associated with heart disease in childhood (i.e., atherosclerosis and left ventricular hypertrophy) but are associated with an increased prevalence of CVD risk factors in adulthood—which results in increased CVD morbidity and mortality (4–8).

These known associations and the now well recognized phenomenon of CVD risk factor tracking from childhood into adulthood has resulted in a greater emphasis on obesity prevention. In addition, the American Heart Association and the American Academy of Pediatrics (AAP) have emphasized the importance of primordial and primary prevention to achieve CVD risk reduction in youth (9, 10). An essential component of this strategy is regular screening for elevated BP and hypertension among children. Current guidelines recommend at least yearly BP measurements in all children 3 years of age and above. Any BP elevation should be confirmed with repeated measurements and the diagnosis of hypertension should be given to any child with a sustained elevation in his/her BP at or above the 95th percentile when measured by manual auscultation. All children with a diagnosis of hypertension should undergo evaluation for a secondary cause (11).

## Evaluation of the Child with Obesity and Hypertension

As introduced above, children with elevated BP should have their BP elevations confirmed with repeated measurements done by manual auscultation. Children with obesity can present unique challenges to BP measurement. For one, their arm size may be sufficiently large to require a BP cuff much bigger than would be expected by the labeling on the cuff (i.e., adult, large adult, thigh). Analyses using National Health and Nutrition Examination Survey (NHANES) data revealed that, based on measured mid-arm circumferences, some children as young as 3–5 years of age require an adult cuff, and starting at age 12 years some children require the use of a thigh cuff for proper BP measurement (12). Individuals with obesity also often have conically shaped arms, with a large circumference proximally that tapers to a much smaller circumference at the cubital fossa. The difference between the proximal and distal upper arm circumferences can be as large as 20 cm, with the average difference being 8.7 cm (13). Finally, while the mid-arm circumference of children with obesity is often larger than might be expected for age, their arm length is not different than would be expected. This leads to an arm length that is disproportionally short for the cuff required for the measured arm circumference. Often the appropriately sized cuff will overlay the cubital fossa, making BP measurement by manual auscultation difficult and raising the possibility of inaccurate measurements.

These challenges are particularly problematic because accurate BP measurement in the child with obesity is perhaps even more important than among those peers of normal or healthy weight. In children up to 20 years of age, obesity is defined as a body mass index (BMI) ≥ 95th age- and sex-specific percentile and overweight is defined as a BMI ≥ 85th percentile but <95th percentile. Severe obesity in children is defined as either a BMI ≥ 120% of the 95th percentile (corresponding to the 99th percentile BMI), or a BMI ≥ 35 kg/m^2^ (corresponding to the cutpoint of Class II obesity in adults), whichever is lower. After age 20 years, adult cutpoints for overweight and obesity apply (BMI between 25 and 30 kg/m^2^ and BMI ≥30 kg/m^2^, respectively). Adult studies have demonstrated that the risk of hypertension increases substantially with increasing BMI, with the odds of hypertension as great as 4.8 among adults with Class III obesity (BMI ≥40 kg/m^2^) when compared to adults with a normal BMI (14). Obesity is also associated with resistant hypertension: hypertensive adults with Class III obesity had a 30% lower odds [OR 0.7, 95% confidence interval (CI) 0.5–0.9] of achieving a BP < 140/90 compared to those with a normal BMI (15).

Once a child with obesity has confirmed hypertension, he/she should undergo an evaluation to investigate for secondary causes of hypertension and screen for CVD risk factors. This evaluation should include the following (11):

**Table:** 

Evaluation	Unique aspects to obesity
Detailed history	Sleep history ○ Daytime somnolence ○ Snoring ○ Witnessed apneic events Diet history ○ Sugar-sweetened beverage intake ○ Fiber intake ○ Total calories consumed ○ Timing and frequency of meals Physical activity ○ Amount and intensity ○ Musculoskeletal pain (which might impact ability to be active) Psychosocial history ○ Depression ○ Anxiety

Detailed physical exam	Anthropometrics ○ Body mass index ○ Waist circumference Skin exam ○ Acanthosis nigricans ○ Hirsutism ○ Striae Abdominal exam ○ Hepatomegaly

Laboratory assessment	Fasting lipids Fasting glucose and insulin Hemoglobin A1c Aspartate transaminase and alanine transaminase

Tests to consider, particularly in children with obesity hypertension:

**Table:** 

Evaluation	Unique aspect to obesity
Polysomnography	Obstructive sleep apnea

Toxicology screen	Depression and anxiety are increased in obese children, leading to increased risk for illicit substances

If a secondary cause is not determined after the initial evaluation in an older child or a child with a BP close to the 95th percentile, additional evaluation may not be warranted. One may then consider a diagnosis of primary or obesity-related hypertension.

## Role of Adiposity on BP in Children

As in adults, BMI influences BP in children. In a large cohort study of more than 100,000 children and adolescents followed for several years, those with obesity and severe obesity had higher BP at baseline and a greater odd of developing hypertension years later than those of lower BMI categories (16). The influence of adiposity on BP in children is also evident in the normative tables utilized by pediatric providers to diagnose hypertension. These tables were developed based on the first manual BP measurement obtained in children enrolled in any of 11 separate studies. The approximately 60,000 healthy children included in this database included children of normal weight as well as those with overweight or obesity. In fact, 21% of the included children had a BMI in the overweight or obese category. The impact of including these children is significant: when overweight and obese children are excluded from the normative table database, BP norms are lower by several mmHg across the board (17). The recently updated pediatric hypertension guidelines have addressed this issue by developing new tables based upon data from youth without overweight/obesity (18).

There are several possible pathophysiological pathways to explain why adiposity is associated with elevated BP and hypertension. The central tenet relates to the dysfunctional adipocyte and neurohormonal activation of the sympathetic nervous system (SNS). It is important to remember that the adipocyte not only serves as a storage depot for fat but is also an active endocrinological cell. Overweight and obesity is associated with a greater mass of adipose tissue, which includes adipocytes as well as pre-adipocytes, macrophages, and fibroblasts among other cell types (19). This tissue secretes various hormones and cytokines, known as adipokines, to maintain homeostasis. In the obese state, adipocytes are greater in number and size, and increasing amounts of adipokines are secreted. Over time, there is upregulation of pro-inflammatory adipokines. When the pro-inflammatory adipokines (leptin, resistin and IL-6, as examples) overwhelm the anti-inflammatory (i.e., adiponectin) adipokines, this imbalance leads to adipose tissue dysfunction and a chronic inflammatory state.

Many of these adipokines lead to an increase in SNS activity. Leptin, for example, has been shown to activate the SNS and studies in humans have demonstrated that Leptin deficiency is associated with lower SNS activity. This Leptin-mediated SNS activation appears to be dependent on the expression of leptin receptors on the proopiomelanocortin neurons in the brain (20). Mice without these receptors are resistant to the hypertensive effects of Leptin (21) and rats given alpha and beta blocker medications were similarly resistant to BP effects of intravenous infusion of Leptin (22).

Sympathetic nervous system activation can impact all organs, but in obesity appears to preferentially impact the renal vascular beds. Increasing BMI in humans is associated with increasing amounts of norepinephrine spill over in the kidneys, suggesting a link between obesity-related SNS activation and the neural release of renin (23). So, in addition to its direct vasoconstricting effects, increased SNS activity also leads to elevated BP and hypertension by increasing renin–angiotensin–aldosterone system (RAAS) activity. RAAS activity increases BP directly (angiotenisin II-mediated vasoconstriction and further SNS activation) and indirectly (angiotensin II- and aldosterone-mediated salt and water tubular reabsorption and ADH-mediated water retention). RAAS activity is further increased with increasing fat mass: adipocytes also secrete RAAS hormones and mineralocorticoid stimulating factors, with the relative contribution of these circulating levels related to the amount of adipose tissue present (24).

In the obese state, there is also increased inflammation with macrophages infiltrating the adipose tissue, and there is increased free fatty acid levels. Dyslipidemia, specifically elevated LDL-cholesterol and triglycerides and low HDL-cholesterol, is frequently comorbid with obesity. Elevated cholesterol is a known CVD risk factor, but its contribution to elevated BP and hypertension is complex. In addition to causing atherosclerosis, elevated LDL-cholesterol induces chronic inflammation, activates the SNS (25), and increases RAAS activity (26, 27). Among hypercholesterolemic individuals treated with statin therapy, SNS activity decreases along with a decrease in LDL-levels (28, 29).

Increased oxidative stress is another significant contributor to obesity-related hypertension. Oxidative stress also promotes SNS activation throughout the hypothalamus. Ultimately, with obesity full metabolic dysfunction occurs, leading to endothelial dysfunction, impaired pressure natriuresis, and poor vascular function, with hypertension the clinically identifiable outcome (30).

The increase in RAAS and SNS activation in obesity-related hypertension is important to recognize, as this has treatment implications for patients with obesity-related hypertension. As mentioned above, the serum levels of almost all components of RAAS are elevated in obesity, and in fact, the amount of adipose tissue present determines the relative amount of circulating angiotensinogen and angiotensin II (24). Weight loss, the first-line treatment for individuals with obesity-related hypertension, leads to a decrease in SNS activity which has direct effects on arterial pressure (decreased peripheral vasoconstriction), indirect effects on arterial pressure (improved pressure natriuresis resulting in lower intravascular volume), and a decrease in renin release from the kidney. In fact, menopausal women who successfully lost 5% of their body weight decreased their SBP by 7 mmHg and had lower angiotensinogen, renin, aldosterone, and angiotensin converting enzyme levels (31), providing experimental evidence for this effective means of treating obesity-related hypertension.

The important role of the SNS in obesity-related hypertension has been demonstrated in animal and human studies. In an experiment comparing the effect of weight gain on BP between dogs with and without bilateral renal denervation, both groups were fed the same high-fat diet and both had the same weight gain after 5 weeks. Despite this, only the dogs with intact renal innervation had higher BP and diminished urinary sodium excretion (32). In adults, adiposity is directly associated with increased muscle sympathetic nerve activity, which is considered a marker of overall sympathetic outflow (33). And, while invasive studies of SNS activity have not been done in children, children with obesity-related hypertension have higher resting heart rates and increased BP variability than non-obese normotensive children (34). Both of these findings are considered indirect markers of SNS activity.

Finally, as with other visceral organs, in the obese state, the kidney can become fat encapsulated (19). When this occurs, there is increased renal interstitial fluid pressure and slower tubular flow rates which ultimately leads to increased sodium reabsorption and increased intravascular volume.

## Treatment Approach to Obesity-Related Hypertension

The primary approach to all children with obesity-related hypertension should focus on the attainment of a healthy weight and achievement of a heart healthy lifestyle. The AAP recommends a staged approach to obesity treatment, with weight loss recommended for children 6 years of age and above when BMI is in the obese category and weight maintenance for growing children when BMI is in the overweight category (35). Weight loss is particularly important for children with obesity-related hypertension because it addresses the underlying etiology, it improves comorbidities and it reduces SNS activation, leading to lowering of BP. Several studies conducted in overweight and obese children ranging from 6 to 16 years of age have demonstrated the effectiveness of weight loss on lowering BP in children. These studies all incorporated diet, physical activity, education, and counseling and demonstrated a decrease in SBP from 6 to 16 mmHg over 5- to 12-month periods of intervention (36–38).

Dietary change is another important aspect of the lifestyle modifications needed to treat obesity-related hypertension. In 2011, the AAP published updated dietary recommendations for the treatment of hypertension (10). Regardless of stage of hypertension or etiology of hypertension, hypertensive children should institute the cardiovascular health integrated lifestyle diet and dietary approaches to stop hypertension (DASH) eating plan, which includes:

– increased intake of fresh vegetables, fruits, and low-fat dairy
– reduced carbohydrate, fat, and processed sugar intake
– limited/avoidance of sugar-sweetened beverages
– encouraged intake of foods with high dietary fiber content (age + 5 = number of grams/day up to 14 g/1000 kcal)

These dietary interventions have been shown to decrease BP in adults. In a randomized controlled feeding trial of adults with pre- or stage 1 hypertension, systolic BP was reduced by 6.6 (95% CI: 4.0–9.1) mmHg when following a DASH diet with a high sodium intake (compared to a usual diet with high sodium intake) and by 8.3 (95% CI: 6.6–10.0) mmHg when following a low-sodium diet in the setting of a Usual diet (compared to a high-sodium Usual diet) among those with hypertension. The degree of BP reduction was even greater when both interventions were implemented simultaneously: systolic BP was reduced by 11.5 (95% CI: 8.9–14.1) mmHg when a low sodium, DASH diet was followed (39) Hypertensive adolescents have also demonstrated decreased BP when enrolled in a clinic-based behavioral nutrition intervention emphasizing a DASH-type diet: systolic BP decreased by 10 mmHg and diastolic BP decreased by 6 mmHg among those prescribed a DASH-type diet (40).

Avoidance of sugar-sweetened beverages can also lead to weight loss among children and has been independently associated with BP reduction in adults. Two randomized controlled trials in children, one of overweight/obese children and another of normal-weight children, each demonstrated that elimination of sugar containing beverages leads to a reduction in weight and measures of adiposity (41, 42). In the PREMIER: Lifestyle Interventions for BP Control trial, a reduction of one 12-oz serving of sugar-sweetened beverages/day among adults over 18 months was associated with a reduction of systolic BP by 1.8 mmHg (95% CI: 1.2–2.4) and diastolic BP by 1.1 mmHg (95% CI: 0.7–1.4). These results remained significant even after adjusting for weight change (43).

There are no current guidelines regarding the degree of sodium reduction in children for the treatment of hypertension. Children should adhere to the Dietary Guidelines for Americans 2015–2020 (44) which states that children ≥14 years should limit daily sodium intake to <2,300 mg and younger children should limit sodium their sodium intake even more, with the upper tolerable limit for children 1–3 years of age being 1,500 mg. These guidelines also state broadly that pre-hypertensive and hypertensive individuals should reduce sodium intake to <1,500 mg. There is evidence to suggest that these recommendations may have a more significant impact on BP among children and adolescents who are overweight/obese than those who are not. In an NHANES study that included 6,235 children 8–18 years of age, 37% of whom were overweight or obese, usual sodium intake was estimated by multiple 24-h recalls. Notably, overall mean sodium intake was much greater than recommendations, with mean intake 3,387 mg/day. Overall, each 1 g of sodium intake per day was associated with an increased systolic BP SD score of 0.121 (95% CI 0.034–0.207) even after adjusting for age, sex, race, and energy intake. However, when the subjects were stratified by weight status, sodium intake was no longer associated with BP among the children of normal weight but it remained significantly associated with BP among the overweight/obese children (each 1 g sodium intake was associated with an increase in systolic BP SDS score of 0.197, 95% CI 0.036–0.357) (45). This differential effect of adiposity on the association of sodium intake and BP may be explained by the activation of the RAAS and SNS in the obese state as detailed above. Future studies need to explore this further.

Other essential components of the Heart Healthy Lifestyle include regular physical activity and a limit to sedentary activities. Specifically, children ≥5 years of age should partake in at least 1 h of moderate-to-vigorous exercise every day and all children should decrease their sedentary activities to <2 h per day. This may be difficult to achieve for children with obesity due to comorbidities such as arthritis, Blount’s disease, slipped capital femoral epiphysis, spinal complications and acute fractures, all of which contribute to decreased mobility. Referral to physical therapy can be helpful for some children as they work to achieve their physical fitness goals.

## Role of Pharmacologic Therapy

While all children with obesity-related hypertension should be prescribed therapeutic lifestyle changes, some children will also require an antihypertensive medication to adequately treat their hypertension. Children with a secondary etiology of hypertension, who are symptomatic, who have diabetes (type 1 or type 2), or who have end-organ damage such as left ventricular hypertrophy should all be prescribed an antihypertensive medication. In addition, children with persistent hypertension after 6–12 months of instituting a heart healthy lifestyle should also be prescribed a medication to lower their BP while they continue to work on weight loss and lifestyle changes (11).

As with any medication, the particular agent chosen should be aimed at treating the underlying etiology, with particular attention being paid to comorbid conditions. As activation of the RAAS system is one of the main ways in which obesity contributes to elevated BP and hypertension, it follows that angiotensin converting enzyme inhibitors (ACEi) or angiotensin receptor blockers (ARB) would be appropriate initial agents in the treatment of obesity-related hypertension. In addition to being able to directly target pathways leading to elevated BP, ACEi or ARB may also have beneficial effects on diabetes and dyslipidemia, two common comorbidities in overweight and obese individuals (46).

Some agents are less ideal in the treatment of obesity-related hypertension. Diuretics, for example, may have similar antihypertensive efficacy as ACEi/ARB in hypertensive, obese adults and may be associated with lower CV events in adults, but they also reduce intravascular volume and cardiac output and stimulate the SNS and RAAS. These agents can also worsen insulin resistance and dyslipidemia and can increase glucose and uric acid levels, particularly in obese individuals. Beta-blockers reduce BP by decreasing both cardiac output and renin activity. However, these agents can lead to weight gain and increased triglycerides and decreased HDL-cholesterol levels. They also have inferior outcomes in adults, particularly when restricted to obese subjects, which may be related to these known side effects (47, 48).

As with non-obesity-related hypertension, the goal of therapy is to lower BP. BP should be treated to below the 90th percentile, or below 130/80 for children ≥ 13 years old (18).

## Conclusion

Obesity-related hypertension is highly prevalent among US children. There are multiple mechanisms by which obesity can lead to elevated BP and increased CVD risk. Central to this is adipose tissue dysfunction, related to an imbalance in the pro- and anti-inflammatory activities of the adipocyte. First-line antihypertensive therapy for children with obesity-related hypertension is weight loss. In addition, instituting a heart healthy lifestyle that includes daily physical activity and a diet rich in fruits and vegetables, fiber and low-fat dairy, that is also low in carbohydrates and sugar-sweetened beverages is essential in the treatment of obesity-related hypertension. There may be challenges to this in children with obesity, related to comorbid conditions such as depression, anxiety, and decreased mobility. A multidisciplinary approach is often required for maximal effectiveness. Some children will require antihypertensive pharmacologic therapy. In children, no agent has been shown to be more effective at lowering BP than another. However, the decreased CV events found among adults treated with RAAS blockers and the adverse metabolic profile associated with beta blocker and diuretic use, make RAAS blockade potentially beneficial in the treatment of pediatric obesity-related hypertension.

## Author Contributions

TB was the sole contributor to the structure of the work and to the acquisition and interpretation of data for the work. TB was solely responsible for drafting the work, provided final approval of the version to be published; and agreed to be accountable for all aspects of the work in ensuring that questions related to the accuracy or integrity of any part of the work are appropriately investigated and resolved.

## Conflict of Interest Statement

The author declares that the research was conducted in the absence of any commercial or financial relationships that could be construed as a potential conflict of interest.

## Abbreviations

AAP, American Academy of Pediatrics; ACEi, angiotensin converting enzyme inhibitor; ARB, angiotensin receptor blocker; BMI, body mass index; BP, blood pressure; CI, confidence interval; CVD, cardiovascular disease; DASH, dietary approaches to stop hypertension; NHANES, National Health and Nutrition Examination Survey; RAAS, renin–angiotensin–aldosterone system; SNS, sympathetic nervous system.
